# Literature based discovery of alternative TCM medicine for adverse reactions to depression drugs

**DOI:** 10.1186/s12859-020-03735-8

**Published:** 2020-10-26

**Authors:** Qing Xie, Kyoung Min Yang, Go Eun Heo, Min Song

**Affiliations:** Department of Library and Information Science, Yonsei University, 50 Yonsei-ro Seodaemun-gu, Seoul, 03722 Republic of Korea

## Abstract

**Background:**

In recent years, Traditional Chinese Medicine (TCM) and alternative medicine have been widely used along with western drugs as a complementary form of treatment. In this study, we first use the scientific literature to identify western drugs with obvious side effects. Then, we find TCM alternatives for these western drugs to ameliorate their side effects.

**Results:**

We used depression as a case study. To evaluate our method, we showed the relation between herb-ingredients-target-disease for representative alternative herbs of western drugs. Further, a protein-protein interaction network of western drugs and alternative herbs was produced, and we performed enrichment analysis of the targets of the active ingredients of the herbs and examined the enrichment of Gene Ontology terms for Biological Process, Cellular Component, and Molecular Function and KEGG Pathway levels, to show how these targets affect different levels of gene expression.

**Conclusion:**

Our proposed method is able to select herbs that are highly relevant to the target indication (depression) and are able to treat the side effects caused by the target drug. The compounds from our selected alternative herbal medicines can therefore be complementary to the western drugs and ameliorate their side effects, which may help in the development of new drugs.

## Background

Adverse drug reactions (ADR) are undesirable and unintended effects which occur in conjunction with the intended therapeutic effects of a drug. To solve this problem, it is important to the pharmaceutical industries to develop methods to predict potential drug side effects to identify causal relationships with the drugs [1]. Serious adverse drug reactions (SADRs) are reported to be the fourth leading cause of death in the United States, after cancer, stroke and cardiac disorder, and the number of patients experiencing severe and fatal ADR in US hospitals has increased significantly [2].

In the past, Traditional Chinese Medicine (TCM) and alternative medicine have been excluded from Western medicine. In recent years, however, they have been widely used along with Western drugs as a complementary form of treatment [3]. Since the 1990s, the number of patients seeking alternative therapies has risen sharply, and more than a third of patients in the United States use Complementary and Alternative Medicine (CAM) [4]. In recent years, more and more scientists have become interested in CAM research, and recent research has shown that it can help in the development of health care [5, 6]. As the demand for TCM has increased, many studies have been conducted to investigate new paradigms for ADR, based on network pharmacology and systems biology [7, 8]. To identify adverse drug reactions, many studies have used network analysis of entities such as chemical compounds, genes, proteins, and diseases, by integrating public resources and building relational databases such as Traditional Chinese Medicine Database(TCM Database) [9], Traditional Chinese medicine integrative database(TCMID) [10], Traditional Chinese medicine systems pharmacology database (TCMSP) [11], TarNet [12]. In addition, many studies aimed at identifying the side effects of drugs have involved computational methods, such as machine learning techniques, for predicting adverse drug reactions [13–20].

Although there has been an explosion in interest in CAM internationally, to the best of our knowledge most studies have focused on developing methods to predict drug side effects. There have been few previous studies that matched alternative herb compounds used in TCM to drugs with side effects discovered by Western medicine. In this study, we developed a method to identify alternative herbs for drugs that cause side effects, by combining TCM and western medicine. We chose depression as a case study, which has many adverse effects such as increasing suicidal thought and behavior, insomnia, and sexual dysfunction. Especially, suicide has been recognized as a serious social problem as suicide rates have increased substantially over the past two decades worldwide [21]. Then we analyzed complementary herbs for Western drugs producing severe side effects. The method we proposed takes into consideration finding candidate drugs which have severe side effects, and calculating the substitutability between a candidate drug and a herb indicated for the same condition.

Drug candidates with significant side effects were identified by calculating a drug/side effect score and a drug/indication score for each pair. The conditions for which these drugs are indicated were translated into Chinese and were retrieved from the CNKI (China National Knowledge Internet). We extracted herb/disease pairs from the abstracts of Chinese scientific publications. Substitutability was calculated between candidate drugs and herbs indicated for the same condition by considering the average proportion of herb per dosage, the average number of side effects of the candidate drugs, and the conditions for which they are indicated. We evaluated our results by analyzing data about Herb-Ingredients-Target-Disease for Nefazodone, the highest ranked drug using our method. We created a protein-protein interaction (PPI) network for Nefazodone and candidate alternative herbs, and conducted gene enrichment analysis pertaining to the active ingredients of the herbs. Our depression case study indicated that alternative herbs can mitigate the side-effects of Western drugs, and can be utilized as CAM for the development of the pharmaceutical industries.

### Related work

The emergence of large-scale data in the fields of medicine and pharmacy enabled new approaches for systems biology and pharmacology. These interdisciplinary approaches, especially those based on network analysis theory, provide opportunities for complementary and alternative application of medication to traditional Western medicine methodology. Many studies have integrated multiple data sources with traditional Eastern medicine as a form of meta-database and used them to discover latent relations among biological entities. A TCM database built by Chen [9] shows more than 20,000 compounds from TCM ingredients as 2D and 3D molecular structures. Ye et al. [22] constructed a curated database for Herb Ingredients’ Targets (HIT) from PubMed abstracts. Xue et al. [10] also built a database of traditional prescriptions, herbs, and compounds, including text-mined drug and gene information from resources such as DrugBank, PubChem, and OMIM. Subsequently, the TCMSP was implemented [11].

These network analysis projects have a common purpose: to find prospective drug candidates or to facilitate the repositioning or repurposing of existing drugs by identifying previously undiscovered interactions. In Korea, the Integrated Bio-Pharmacological Network Database for Traditional Korean Medicine, which proposed an established network of traditional Korean medicines, drugs, proteins, indications and side-effects for drug discovery, was published [23], Jeong et al. [24] conducted literature-based research into the clustering of anti-cancer drugs and network analysis of those drugs and target proteins, focusing on pancreatic cancer. A link analysis of compound-target proteins from a semantic network constructed using text-mining data was studied by Fu et al. [25]. Zhang et al. [26] proposed a network-topological similarity-based classification method for the prediction of the association between drugs and diseases. Specifically regarding the prediction of side effects and ADEs, Cheng et al. [27] carried out text mining and constructed a meta-database including known compound-ADE associations, and reported a network model for the prediction of potential ADEs.

As the number of databases containing rich information about chemical compounds, genes, proteins and diseases has increased, many computational methods have been developed for predicting the side effects of drugs, based on this information, before they are released to the market. These prediction and identification studies into ADRs primarily use machine learning techniques, ranging from naive Bayesian models for rapid assessment [17] to support vector machines (SVMs), with or without other techniques [13, 14], to multiple techniques including ensemble or hybrid learning [16, 19, 20, 28], and more complicated algorithms are continuously being developed [29, 30].

In the present study, we used Western medicine databases and traditional literature regarding Chinese medicine for text mining in order to find complementary or alternative medicines for known drugs with significant side-effects. We also tracked the associations of drugs with conditions and side-effects, into which previous research has not been conducted. In the selection of alternative traditional prescriptions, we aimed to maximize the side-effect mitigating efficacy of the herbs.

## Methods

### Research overview

**Fig. 1:**
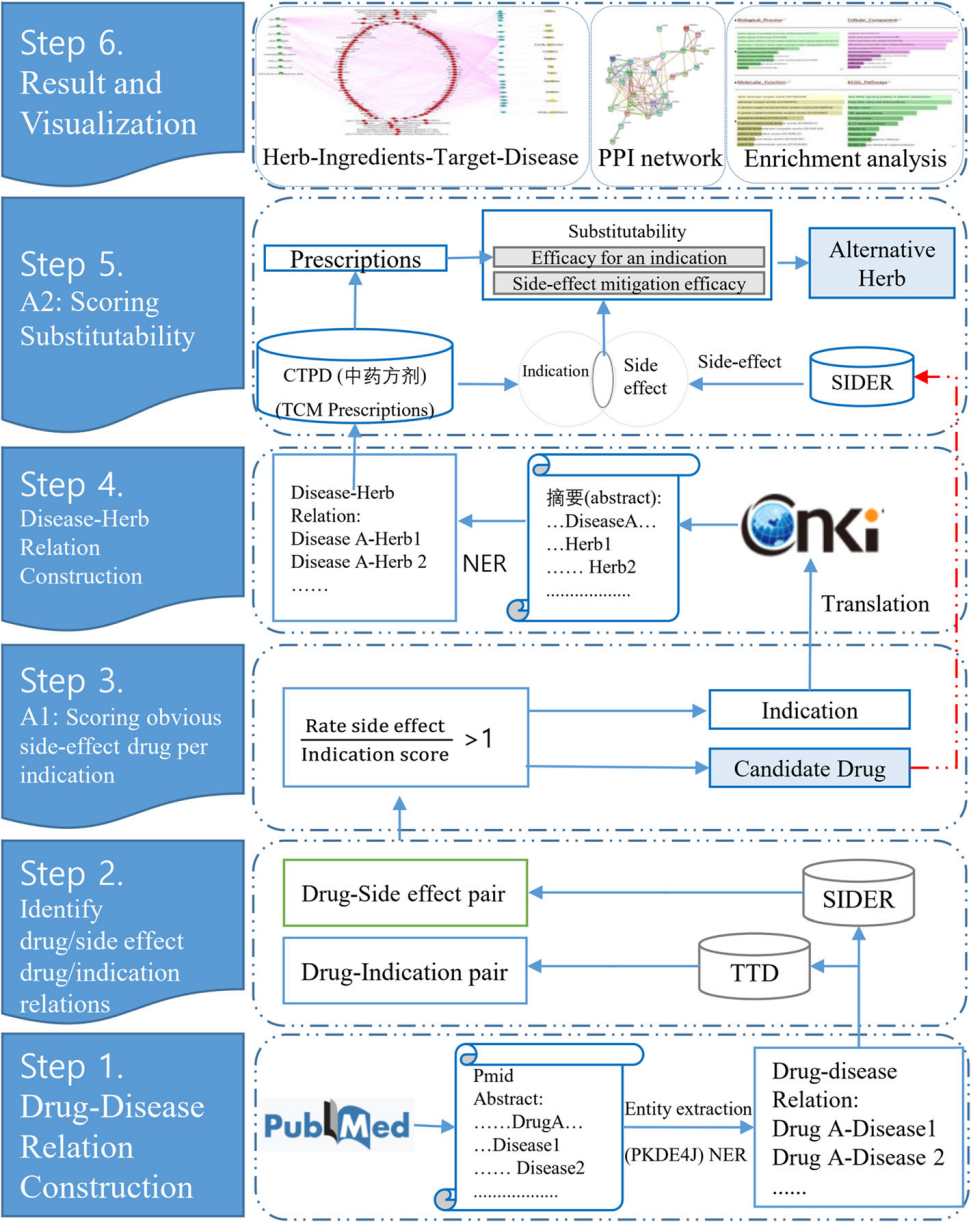
The overall research design

Figure 1 illustrates the overall research design used to explore alternative herbs for drugs with side effects identified in cross-lingual scientific databases. In the initial step, scientific papers were obtained from PubMed, and 169,766 records were collected from 2010 to 2014 (http://informatics.yonsei.ac.kr/download/pubmed2010-2014.txt). We preprocessed the PubMed records and extracted entities using the Named Entity Recognition (NER) technique of PKDE4J [31]. Then we linked drug and disease entities according to their co-occurrence in a single abstract. We then filtered them by database to identify drug/side effect and drug/indication relations. For this, we used SIDER (http://sideeffects.embl.de/download/) to identify drug/side effect relations, and the Therapeutic Target Database (TTD) (http://db.idrblab.net/ttd/) to identify drug/indication relations. We developed an algorithm to select drugs in PubMed which have obvious side effects. Further, we translated the indication for which the drugs were indicated into Chinese and searched for them in the Chinese science database (CNKI) in the domain of traditional Chinese pharmacology. In the same manner, we extracted entities—herb and disease—and linked them by co-occurrence in one abstract. We calculated the substitutability of herbs and drugs indicated for the same condition using the Chinese Traditional Prescriptions Database (CTPD). In the result part, we show the relation between herb-ingredients-target-disease for representative alternative herbs. We also produced a protein-protein interaction network of Western drugs and alternative herbs and we performed enrichment analysis of the targets of the active ingredients of the herbs.

### Selection of drugs to be replaced

In this paper, we calculated the drug/side effect score and drug/indication score using Formulae 1 and 2. Link weight is the drug/side effect or drug−/indication co-occurrence frequency.

1 $$ \mathrm{Side}-\mathrm{effect}\ \mathrm{score}=\frac{target\ side- effect\kern0.5em link\ weight}{average\ side- effect\ link\ weight}\kern0.5em $$

2 $$ \mathrm{Indication}\ \mathrm{score}=\frac{target\ indication\ link\ weight}{average\ indication\kern0.5em link\ weight}\kern0.5em $$

For every drug, we recorded the link weight of the co-occurrence side effects, and then used the target side effect link weight divided by the average side effect link weight. We calculated indication in the same way. If a drug is popular in the scientific field, the side effects and indications of that drug will be better documented than those of a less popular drug. Thus, we used two approaches to reduce the problem of bias in the literature: division by the average side effect or indication; and the use of average side effect score divided by indication score to rank the result.

The assumption of the proposed algorithm is that in the literature, if a drug has low toxicity, the conditions for which that drug is indicated will be mentioned more in publications than those of other drugs which have similar targets. A high indication score may therefore be related to low toxicity. Conversely, if a drug has high toxicity, the side effects of that drug will generally not be in common with those of other drugs. To evaluate the performance of the proposed algorithm based on Formula 1 and 2, we matched the ranked results with the toxicity and half-life indicators of the target drug in DrugBank.

As shown in Algorithm 1, in our dataset, for each drug, we calculated the side effect and indication scores using Formulae 1 and 2. A score of side-effect/indication of more than 1 indicated that this drug may need an alternative in a corresponding indication.

#### Algorithm 1.

Finding obvious side-effect drugs in collected dataset.

**Table:** 

For each drug in dataset:	
Identified drug-side effect and drug- indication relations:	
For each side effect(α is the number of side effects):	
drug- side effect frequency: *f*_*s*_	
side effect score = *f*_s_ / average_side effect_	
*Rate*_se_= ∑side effect score /α	
For each indication:	
drug- indication frequency: *f*_*i*_	
indication score= *f*_*i*_ / average_indication_	
If $\frac{Rate_{\mathrm{se}}}{\mathrm{indication}\ \mathrm{score}\ }$ > 1:	
This drug may need be replaced with alternative medicines	

Eighty-four drugs were identified as having obvious side effects in our dataset.

### Herb data collection

These eighty-four drugs are indicated for sixty-six different indications, according to the drug/indication pairs in our dataset. We translated these indications into Chinese and searched in the CNKI (http://www.cnki.net/). We collected Chinese literature from the Chinese database because the literature on TCM published in English are insufficient due to the low recognition of TCM in Western countries, and we needed to find alternative herbs for the drug, based on traditional Chinese medical prescriptions.

Table 1 shows the top 20 indication names ranked by number of papers. The full indication names are shown in Additional file 1. A total of 47,103 papers were collected from the domain of traditional Chinese pharmacology.

**Table 1:** Chinese data collection

Rank	Indication name		No. of Papers	Rank	Indication name		No. of Papers
	English	Chinese			English	Chinese	
1	arthritis	关节炎	5307	11	rheumatoid arthritis	类风湿性关节炎	1541
2	pain	疼痛	3578	12	pneumonia	肺炎	1428
3	diabetes	糖尿病	3554	13	diabetic nephropathy	糖尿病性肾病	1305
4	hypertension	高血压	3008	14	diarrhea	腹泻	1303
5	asthma	哮喘	2911	15	constipation	便秘	1243
6	blood pressure	血压	2472	16	seizures	癫痫	1073
7	lung cancer	肺癌	2207	17	acne vulgaris	寻常痤疮	948
8	breast cancer	乳腺癌	1690	18	angina pectoris	心绞痛	927
9	heart failure	心力衰竭	1674	19	insomnia	失眠	827
10	major depression	抑郁症	1618	20	diarrhea	痢疾	796

After the Chinese abstracts were collected, we extracted the disease and herb entities from them. The disease and herb names came from a Chinese medicinal materials database, which includes 60,993 records of herb names and indications. The data came from the National Chinese herbal medicine compilation, a Chinese medicine dictionary, the Chinese herb and other sources. We obtained 153,595 disease-herb pairs linked by co-occurrence in one abstract.

### Substitutability

The substitutability of a herb for a drug, which shares a specific indication with the herb, is calculated by considering a) average dosage proportion of a herb D _(Herb)_, and b) the average number of elements of the intersection of side-effects of the drug and the indications of the herb’s prescription (N) as shown in Formula (3).

3 $$ {\mathbf{Substitutability}}_{\left(\mathrm{Drug},\mathrm{Herb}\right)}={\mathrm{D}}_{\left(\mathrm{Herb}\right)}\times \mathrm{N} $$

To calculate the intersection number (N) of side effects produced by a target drug and the indication of the target herb’s prescription, we translated the side effects of the target drug into Chinese. Prescriptions came from the Chinese Traditional Prescriptions databases (CTPD中药方剂), which includes more than 1000 famous medical books including 84,212 prescriptions with prescription composition, source, preparation method, efficacy, usage, and notice.

**Fig. 2:**
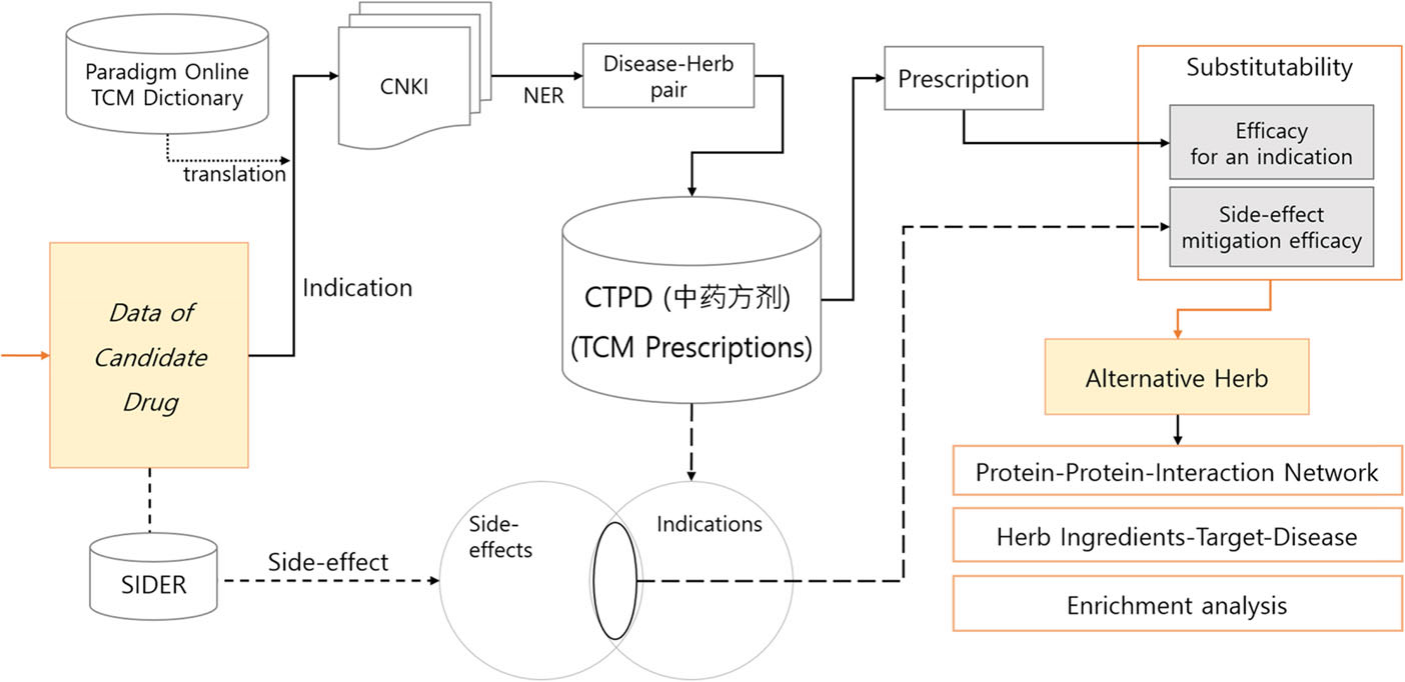
Substitutability calculation and evaluation

Figure 2 shows the substitutability calculation and evaluation process. First, we translated the condition for which a candidate herb is indicated, searched the CNKI, and extracted the disease-herb pair. We searched for these herbs in the CTPD to calculate the average dosage proportion of the target indication and the average number of elements in the intersection of drug side-effects and the indications of the herb’s prescription. Then we took the top 10 herbs ranked by substitutability as alternative herbs for the target drug. Finally, we used the herb-ingredients-target-disease relations to evaluated our result.

Table 2 shows an example. The target drug is mirtazapine, the target herb is 白芍, and the number of intersections of the prescription 1 indication set and the side effect set is three (agitation(烦躁), dizziness (头晕), dry mouth(口燥)). The intersection number of the prescription 2 indication and the side effect set is three (anxiety(忧), vomiting (呕), dizziness(头眩)). *N* is the average number of *N*_*n*_, and then 1 is added, because depression is also an indication in the prescription. In Table 2, the asterisk (*) in the prescription indications column shows common indications with the side effects of the target drug and the dot (•) in bold is the indication (depression).

**Table 2:** Substitutability for mirtazapine and 白芍 (PAEONIAE RADIX ALBA)

Drug- Herb	Drug-side effect	Prescription indications	N_**n**_	Prescription	Dosage
mirtazapine -白芍white peony root s	insomnia (不寐) agitation(烦躁)^a^ anxiety(忧) ^a^ erectile dysfunction(经隧失职) nausea(恶心) vomiting (呕, 吐)^a^ arrhythmia(心悸) bradycardia dizziness (头昏, 头眩)^a^ dry mouth(口燥)^a^ orthostatic hypotension hallucination (妄见)tachycardia(心跳过速)	Prescription 1: 经闭气郁证, 肝郁气滞, 经闭不行, 面色青黄, 精神**抑郁**^b^, 烦躁^a^性急, 头昏^a^耳鸣, 胸胁作胀, 食少嗳气, 舌尖红, 口燥^a^, 脉弦数或弦紧。	3	Prescription 1: 当归2钱, 白芍3钱, 柴胡2钱, 茯苓3钱, 薄荷1钱, 丹皮2钱, 山栀仁2钱, 白术3钱, 泽兰叶4钱, 郁金2钱, 甘草1钱	0.12
		Prescription 2: 暴怒伤肝, **抑郁**^b^忧^a^思, 致肝火妄动, 发为鬓疽, 头眩^a^, 痛彻太阳, 胸膈痞连两胁, 呕^a^酸水。	3	Prescription 2: 当归1钱, 白芍1钱, 茯苓1钱, 白术1钱, 贝母1钱, 熟地1钱, 山栀1钱, 半夏6分, 人参6分, 柴胡6分, 丹皮6分, 陈皮6分, 香附6分, 川芎6分, 甘草4分	0.086
Average			3		0.103

^a^ common indications

^b^ indication (depression)

Before calculating the importance of each herb, we needed to unify the unit of measurement. In our prescription dataset, 1两 (Liang) = 10钱 (Qian) = 100分 (Fen), so we unified all to a minimum unit (Fen).

We calculated the proportion of herb per dosage in Traditional Chinese Medicine prescriptions. In prescription 1, the dosage of 白芍 (PAEONIAE RADIX ALBA) is 3 钱(Qian, the dosage of whole prescription 1 is 25 钱 (Qian), therefore, the importance of the target herb in prescription 1 is 3/25 = 0.12. The average dosage of the target herb in prescription 2 is 0.086. The substitutability of mirtazapine and 白芍 is (3 + 1) × 0.103 = 0.412. If herb and target indication showed in one prescription (direct herb), we used the above method to calculate their substitutability. However, If the herb and the target indication did not show in one prescription, we called them indirect herbs. We calculated the co-occurrence proportion of indirect and direct herbs and then multiplied by the directly connected herb dosage proportion to obtain the indirect herb dosage proportion as the score. We also accumulated all related direct herb scores as the indirect herb dosage proportion. *N*_*indirect*_ is the average number of the intersections of side effects produced by the target drug and the indication of the target herb’s prescription. Algorithm 2 shows the calculation of substitutability for these two cases.

#### Algorithm 2.

Substitutability calculation.

**Table:** 

Chinese Traditional Prescriptions Database. (CTPD) For each drug, creates a side effect set and translates into Chinese. (SE _Chinese_)	
For each herb co-occurrence with the target indication:	
Search the prescriptions with the target indication in CTPD:	
Prescription composition set as P	
Prescription indication set as I	
For each P	
Calculated the percentage of herb in prescription (P _herb_)	
Sum _D_ = ∑ P _herb_	
D _(herb) =_ Average (Sum _D_) For each I:	
N _n_ = SE_(Chinese)_ **∩** Indication	
Sum _n_ = ∑ N _n_	
**N = Average (**Sum _n_) + 1	
If Herb and target indication not show in one Prescription (new relation):	
Prescription indication set as I	
We named herb as herb _indirect_	
For each herb related with target indication in one Prescription:	
F_c_ = herb _indirect_ co-occurrence with herb	
F _h_ = The occurrence number of herb _indirect_ in CTPD	
Percentage _(herb_indirect)_ = (F_c_/ F_h_) × Percentage _(herb)_	
Sum _D_ = ∑ Percentage _(herb_indirect)_	
D _(herb_indirect) =_ Sum _D_	
For each I:	
N _indirect_ = SE_(Chinese)_ **∩** Indication	
Sum _n_ = ∑ N _indirect_	
**N**_**indirect**_ **= Average (**Sum _n_)	
**Substitutability**_(Drug, Herb)_ = D _(herb)_ × N	
**Substitutability**_(Drug, Herb_indirect)_ = D _(herb_indirect)_ × N _indirect_	

## Results

### Evaluation of obvious side effect drugs

We evaluated all drugs in our dataset by toxicity and half-lives of drugs from DrugBank. In use of toxicity, we used the oral LD50 in rat. We did not calculate toxicity if the oral LD50 in rat of those drugs are not founded in the DrugBank database. In addition, if the LD50 and half-life are time buckets, we used the median value. LD50 is the amount of a toxic agent that is sufficient to kill 50% of a population of animals within a certain time. A smaller number means the toxicity is higher. Half-life represents drug persistence. If drug A and B have similar toxicity but the half-life of A is much longer than that of B, the dosing interval of drug A is longer. Table 3 shows the averaged toxicity and half-life of selected and unselected drugs, respectively.

**Table 3:** The averaged toxicity and half-lives of selected and unselected drugs

Type	Averaged Toxicity (mg/kg)	Averaged half-life (hour)
Selected obvious side effect drugs	1667.57	10.87
Unselected drugs	2609.07	12.58

The averaged oral LD50 in rat for unselected drugs was 1.56 times higher than that of selected obvious side effect drugs, which indicated that the selected obvious side effect drugs generally have higher toxicity. In addition, the averaged half-lives of unselected drugs is longer than that of selected obvious side effect drugs.

### Case study: depression

In this section, we describe the result of alternative herbs for obvious side effect drugs in the dataset by dividing the result of algorithm 1 (finding obvious side-effect drugs for depression in the dataset) and algorithm 2 (finding alternative herbs) using depression as the indication. Also, to evaluate our methodology, we show the relation between herb-ingredients-target-disease for salient alternative herbs. We also produced a protein-protein interaction network of Western drugs and alternative herbs, and performed enrichment analysis of the targets of the active ingredients of the herbs. The enrichment of Gene Ontology terms for Biological Process, Cellular Component, and Molecular Function and KEGG Pathway levels, to show how these targets affect different levels of gene expression, was examined.

#### Finding obvious side-effect drugs for depression

In our dataset, there are 12 different kinds of Western drugs for treating depression. However, 7 of them have side effect scores greater than the indication score defined in Algorithm 1. In Table 4, the replaceable score is the side effect score divided by the indication score. A higher score means this drug has a higher need for finding alternative herb medicines in the collected dataset.

**Table 4:** Anti-depression Western drugs

Drug name	Side effect score	Indication	Replaceable Score	DRUGBANK Matching	
				Toxicity	Half life
Nefazodone	0.33333	0.0794702	4.1944025	Cases of life-threatening hepatic failure have been reported in patients treated with nefazodone.	2–4 h
Milnacipran	0.62295879	0.1589404	3.9194490	Oral LD_50_ rat: 213 mg/kg	6–8 h
Mianserin	0.34600026	0.1192	2.9026867	Oral LD_50_ rat: 780 mg/kg	10–17 h
Trazodone	0.81612163	0.31788079	2.5673826	Oral LD_50_ rat: 690 mg/kg	7.3 +/− 0.8 h.
Nortriptyline	0.3283245	0.1589404	2.0657082	Oral LD_50_ rat: 405 mg/kg	26 h
Duloxetine	0.99170246	0.91390728	1.0851237	Fatalities have been reported with doses of 1000 mg involving both mixed drugs as well as duloxetine alone	12 h
Clomipramine	0.41045464	0.39735099	1.03297751	One death involved a patient suspected of ingesting a dose of 7000 mg. The second death involved a patient suspected of ingesting a dose of 5750 mg.	32 h
Don’t need to find Alternative Herbs					
Reboxetine	0.36668174	0.47682119	0.76901309	Reports of seizures (rare) have been reported	12.5 h
Mirtazapine	0.59422385	0.95364238	0.62310972	Oral LD_50_ rat: 830 mg/kg in male Swiss mice	20–40 h
Venlafaxine	0.81547802	1.43046358	0.57007954	Overdose of venlafaxine is typically associated with mild symptoms	5 h
Citalopram	0.92540653	1.82781457	0.50629125	Oral LD_50_ rat:179 mg/kg	35 h
Fluoxetine	0.86558296	5.16556291	0.16756798	In a report that included 234 fluoxetine overdose cases, it was concluded that symptoms resulting from fluoxetine overdose were generally minor and short in duration	1–3 days

Table 4 shows the 7 drugs used as candidates among the 12 drugs which have the target indication of depression. Nefazodone ranked first in our dataset. We examined the toxicity and half-life indicators in DrugBank, and the half-life of Nefazodone is only two to four hours.

There were five drugs that there is no need to find alternative herb medicines (Table 4). For example, reboxetine and mirtazapine have low toxicity. Venlafaxine’s half-life is short, but the toxicity is lower. Canadian clinical practice guidelines recommend venlafaxine as a first-line option for the treatment of depression [32]. Citalopram has a certain level of toxicity, but its half-life is longer than that of the other drugs. Fluoxetine has many side effects, but none of them are serious. Its half-life is the highest of all of the anti-depression drugs. Thus, fluoxetine appears to be the most suitable drug for the treatment of depression in our dataset.

In the literature, if a drug has low toxicity, the conditions for which that drug is indicated will be mentioned more in publications than those of other drugs which have similar targets. A high indication score may therefore be related to low toxicity. Conversely, if a drug has high toxicity, the side effects of that drug will generally not be in common. For example, headache is a side effect, but is very common in many drugs, so the side effect score these drugs is not higher in our algorithm. A higher side effect score may be related to higher toxicity. Finally, we used the side effect score divided by the indication score to strengthen the difference.

#### The alternative herbs for obvious side-effect drugs for depression

In Algorithm 2, we used the average proportion of the target herb dosage in the target indication (depression) prescriptions and the average number of elements of the intersection of side-effects of the drug and the indications of the herb’s prescription. We named this metric ‘substitutability’.

However, not all herb/disease pairs as indicated by co-occurrence in the literature are directly connected in prescriptions. For these herbs, we could not directly calculate the average dosage proportion. In the Traditional Chinese Prescription Database (TCPD), there are 57 prescriptions related to depression. After relation extraction, we identified 258 herbs which co-occurred with depression, and 97 herbs co-occurred with depression in prescriptions. For the 161 herbs which are not directly connected in prescriptions, we first calculated the co-occurrence proportion of these herbs and directly connected herbs as shown in the lower part of Algorithm 2. We then multiplied this measure by the directly connected herb dosage proportion to calculate indirect herb dosage proportion. Thus, we calculated the substitutability of all anti-depression drug and herb medicines.

In Table 5, the No. 1 herb in the Nefazodone-related list is 藿香 (*POGOSTEMON CABLIN* BENTH). TCMSP shows *POGOSTEMON CABLIN* BENTH including quercetin, which is used to treat depression, insomnia, asthma, gout, and arthritis. Among these indications, depression is the indication of Nefazodone. The side effects of Nefazodone are insomnia, asthma, gout, and arthritis, so the ingredients of *POGOSTEMON CABLIN* BENTH can treat the side effects of Nefazodone.

**Table 5:** Drugs and alternative herbs for depression

Rank	Drug	Herb (English name)	Score	Herb (English name)	Score
1	Nefazodone	藿香(*POGOSTEMON CABLIN* BENTH)	0.4615	丹参(RADIX SALVIAE)	0.2667
		桃仁(PERSICAE SEMEN)	0.4528	黄柏(CORTEX PHELLODENDR)	0.2558
		紫苏(*PERILLA FRUTESCENS*)	0.4030	熟地(REHMANNIAE RADIX PRAEPARATA)	0.2520
		红花 (CARTHAMI FLOS)	0.3396	青皮(CIRTRI RETICULATAE PERICARPIUM VIRIDE)	0.2374
		柏子仁(PLATYCLADI SEMEN)	0.2667	白芍(PAEONIAE RADIX ALBA)	0.2374
2	Milnacipran	藿香(POGOSTEMON CABLIN BENTH)	0.5769	红花 (CARTHAMI FLOS)	0.3962
		紫苏(PERILLA FRUTESCENS)	0.5543	熟地(REHMANNIAE RADIX PRAEPARATA)	0.3528
		桃仁(PERSICAE SEMEN)	0.5283	夏枯草(PRUNELLAE SPICA)	0.3
		柏子仁(PLATYCLADI SEMEN)	0.4	乌药(LINDERA RADIX)	0.2696
		丹参(RADIX SALVIAE)	0.4	肉桂(CINNANMOMI CORTEX)	0.2667
3	Mianserin	紫苏(PERILLA FRUTESCENS)	0.7054	丹参(RADIX SALVIAE)	0.4
		藿香(POGOSTEMON CABLIN BENTH)	0.6923	芍药(PAEONIAE LACTIFLORA)	0.3885
		桃仁(PERSICAE SEMEN)	0.6038	茯苓(PORIA COCOS)	0.3654
		红花 (CARTHAMI FLOS)	0.4528	三七(PSEUDO GINDENG)	0.3636
		柏子仁(PLATYCLADI SEMEN)	0.4	五灵脂(TROGOPTERUS DUNG)	0.3636
4	Trazodone	藿香(POGOSTEMON CABLIN BENTH)	0.5769	乌药(LINDERA RADIX)	0.2696
		紫苏(PERILLA FRUTESCENS)	0.5543	肉桂(CINNANMOMI CORTEX)	0.2667
		桃仁(PERSICAE SEMEN)	0.3774	柏子仁(PLATYCLADI SEMEN)	0.2667
		芍药(PAEONIAE LACTIFLORA)	0.3237	丹参(RADIX SALVIAE)	0.2667
		红花 (CARTHAMI FLOS)	0.2830	青皮(CIRTRI RETICULATAE PERICARPIUM VIRIDE)	0.2612
5	Nortriptyline	藿香(POGOSTEMON CABLIN BENTH)	0.5769	肉桂(CINNANMOMI CORTEX)	0.2667
		紫苏(PERILLA FRUTESCENS)	0.5543	柏子仁(PLATYCLADI SEMEN)	0.2667
		桃仁(PERSICAE SEMEN)	0.3774	丹参(RADIX SALVIAE)	0.2667
		熟地(REHMANNIAE RADIX PRAEPARATA)	0.3024	青皮(CIRTRI RETICULATAE PERICARPIUM VIRIDE)	0.2612
		红花 (CARTHAMI FLOS)	0.2830	芍药(PAEONIAE LACTIFLORA)	0.2374
6	Duloxetine	桃仁(PERSICAE SEMEN)	0.5283	乌药(LINDERA RADIX)	0.2696
		红花 (CARTHAMI FLOS)	0.3962	柏子仁(PLATYCLADI SEMEN)	0.2667
		藿香(POGOSTEMON CABLIN BENTH)	0.3462	丹参(RADIX SALVIAE)	0.2667
		熟地(REHMANNIAE RADIX PRAEPARATA)	0.3024	赤芍(RADIX PAEONIAE RUBRA)	0.2642
		紫苏(PERILLA FRUTESCENS)	0.3023	青皮(CIRTRI RETICULATAE PERICARPIUM VIRIDE)	0.2374
7	Clomipramine	紫苏(PERILLA FRUTESCENS)	0.7054	柏子仁(PLATYCLADI SEMEN)	0.4
		藿香(POGOSTEMON CABLIN BENTH)	0.6923	丹参(RADIX SALVIAE)	0.4
		桃仁(PERSICAE SEMEN)	0.5283	红花 (CARTHAMI FLOS)	0.3962
		乌药(LINDERA RADIX)	0.4494	芍药(PAEONIAE LACTIFLORA)	0.3669
		熟地(REHMANNIAE RADIX PRAEPARATA)	0.4032	茯苓(PORIA COCOS)	0.3654

#### Relation of herb-ingredients-target gene or protein-disease(side effects)

**Fig. 3:**
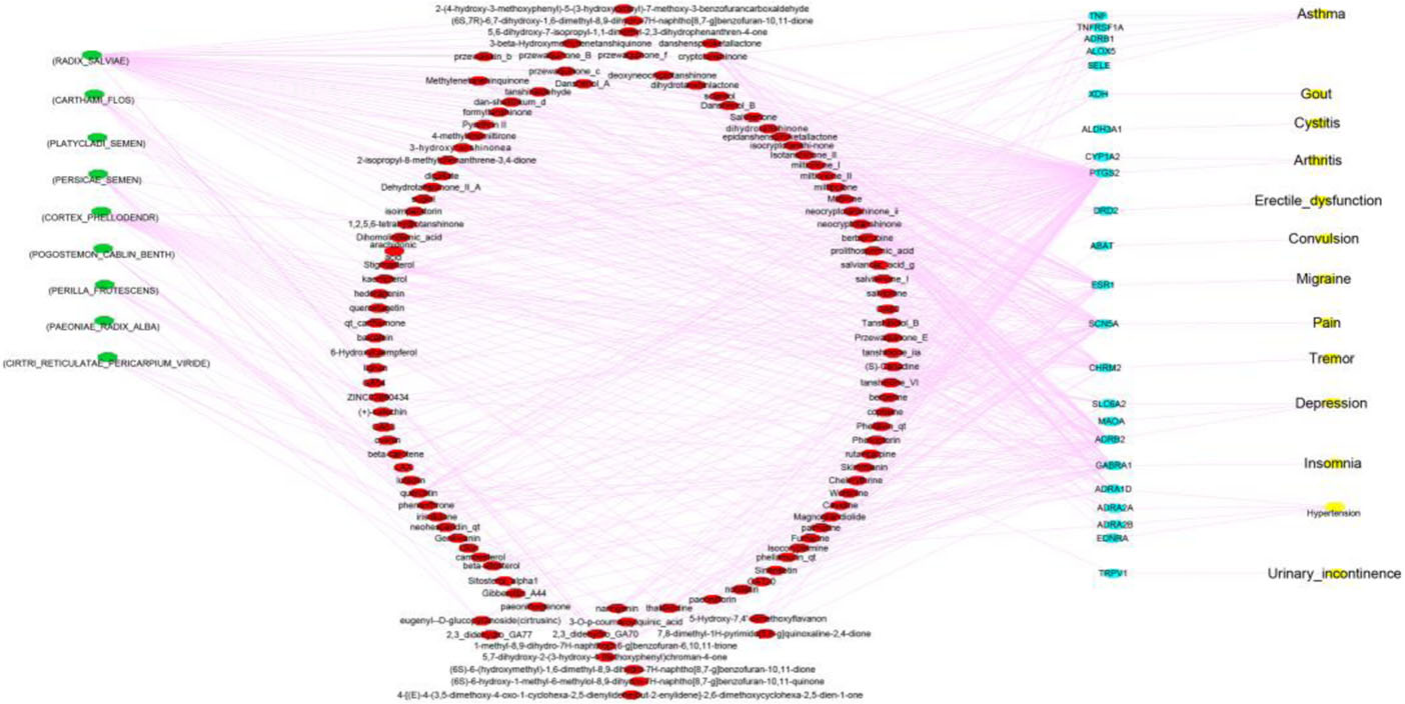
Herb ( )- Ingredient( ) -Target gene or protein( ) -Disease*( ) *Disease is a side effect or an indication of Nefazodone

As shown in Table 5, the Nefazodone related herb list includes POGOSTEMON CABLIN BENTH, PERSICAE SEMEN, PERILLA FRUTESCENS, CARTHAMI FLOS, PLATYCLADI SEMEN, RADIX SALVIAE, CORTEX PHELLODENDR, REHMANNIAE RADIX PRAEPARATA, CIRTRI RETICULATAE PERICARPIUM VIRIDE, PAEONIAE RADIX ALBA. The ingredients of the top 10 herbs are related to Nefazodone. For the herb ingredients, we selected oral bioavailability (OB) greater than 30%, and drug-likeness (DL) greater than 0.18. Figure 3 shows herb-ingredient-target gene or protein-disease relations. In this figure, we show only Nefazodone-related diseases involving side effects or indications of Nefazodone.

We choose Nefazodone because it is the highest-ranked drug (Table 4). The relation of herbs and ingredients was matched using TCMSP, while ingredients and diseases were matched using DrugBank. Nefazodone shows 206 side effects, in other words, diseases in SIDER, and 13 diseases including gout, asthma, and migraine are matched with what the herb ingredients could treat (OB > 30, DL > 0.18). These diseases in Fig. 3 are the side effects. Nefazodone is an anti-depression drug, and the target genes/proteins of herb ingredients such as SLC6A2, ADRB2, MAOA are related to depression disorder. Stigmasterol from 红花(CARTHAMI FLOS) (4th for Nefazodone) targets these three genes or proteins, which means our selected herb not only has the same indication (depression) as the Western drug and but also could mitigate the side-effects of that drug.

Figure 3 shows the implications of herb substitutability, which means the herbs of similar curative effect of the Western medicine have the ability to reduce the side-effects of it at the same time. Our depression-focused study could be generalized to other cases for discovering complementary compounds from alternative herbal medicines.

#### Protein-protein interaction network Nefazodone and its alternative herbs

Table 6 shows the target of Nefazodone (DrugBank). We used the targets from Nefazodone and main active alternative herb ingredients (blue dots in Fig. 3) to create a protein-protein interaction network.

**Table 6:** Target proteins of Nefazodone

TARGET	STMBOL	ACTIONS
A5-hydroxytryptamine receptor 2A	HTR2A	antagonist
A5-hydroxytryptamine receptor 2C	HTR2C	antagonist
ASodium-dependent serotonin transporter	SERT	inhibitor
A5-hydroxytryptamine receptor 1A	HTR1A	antagonist
ASodium-dependent noradrenaline transporter	SERT	inhibitor
USodium-dependent dopamine transporter	SLC6A3	inhibitor
UAlpha-1B adrenergic receptor	ADRA1B	other/unknown
UAlpha-2A adrenergic receptor	ADRA2A	antagonist
NAlpha-1A adrenergic receptor	ADRA1A	antagonist
UPotassium voltage-gated channel subfamily H member 2	KCNG1	antagonist

**Fig. 4:**
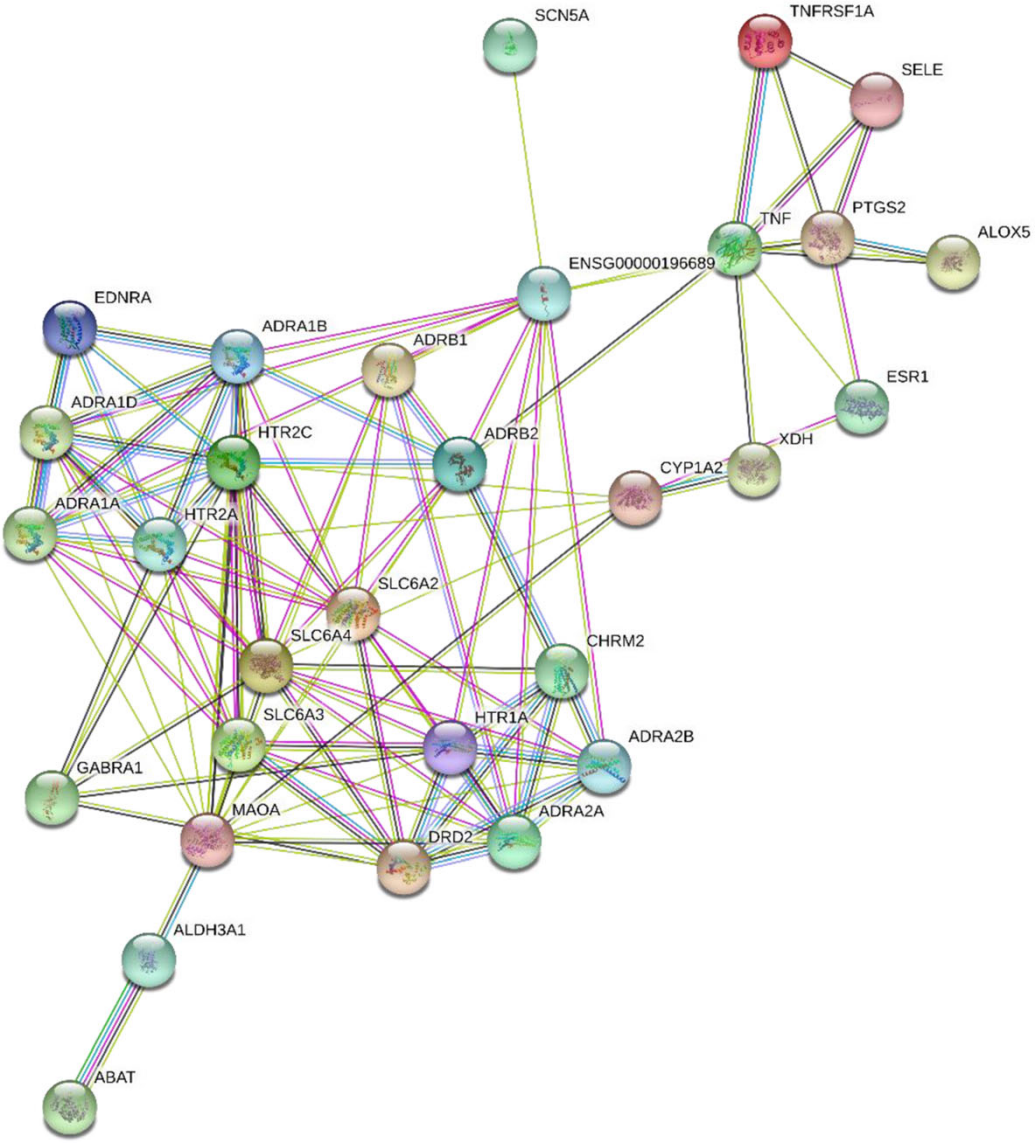
Protein-protein interaction network of Nefazodone and alternative herbs. The edges means interactions of proteins, including ( ) from curated databases, ( ) experimentally determined, ( ) gene neighborhood, ( ) gene fusions, ( ) gene co-occurrence, ( ) text mining, ( ) co-expression, ( ) protein homology

Figure 4 is created by STRING (https://string-db.org/). HTR2A is a protein which functions as a receptor for various drugs and psychoactive substances, Nefazodone acts on HTR2A and has palliative effect on depression. Via co-expression, protein homology, gene co-occurrence, and text mining, we found that this protein could have interactions with ADRA1D, and it is an alpha-adrenergic receptor which mediates its effect through the influx of extracellular calcium. ADRA1D comes from the herb 丹参 (RADIX SALVIAE), which is effective on hypertension.

In Fig. 4, MAOA is found related to HTR2A, ADRA2A by text mining, and to HTR2C, ADRA1B by co-expression with Nefazodone. MAOA is involved in the breakdown of the neurotransmitter serotonin. Signals transmitted by serotonin regulate mood, emotion, sleep, and appetite, which help treatment of depression. MAOA comes from stigmasterol, an ingredient of herb 红花(CARTHAMI FLOS). These relations show that alternative herbs could help the Western drug to improve the effectiveness of the treatment at the gene or protein level.

#### Enrichment analysis

**Fig. 5:**
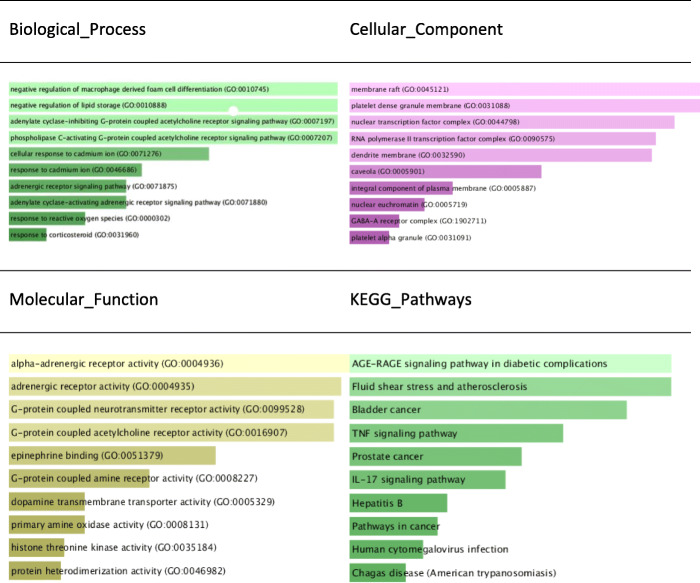
Enriched Gene Ontology terms for Biological Process, Cellular Component, and Molecular Functions, and KEGG Pathways of targets from main active ingredients of alternative herbs, ranked by *P*-value

We used all 221 target genes or proteins of alternative herbs for gene enrichment analysis. Figure 5 shows the enriched Gene Ontology terms for biological process, cellular component, and molecular function, and KEGG Pathways of target genes/proteins from the main active ingredients of alternative herbs ranked by *P*-value.

#### The relations of candidate drugs and their alternative herbs

In Table 7, we show the common target proteins between other western drugs (Table 5) and their alternative herbs. The targets of western drugs come from DrugBank. The relations of herbs, ingredients and targets were matched using TCMSP.

**Table 7:** The common target of drugs and their alternative herbs

*Drug name*	Common Target	Alternative Herb (ingredients)
Milnacipran	Sodium-dependent serotonin transporter	桃仁(beta-sitosterol), 紫苏(beta-sitosterol), 红花(beta-sitosterol), 夏枯草(beta-sitosterol) 乌药(beta-sitosterol, Boldine), 藿香(5-Hydroxy-7,4′-dimethoxyflavanon)
	Sodium-dependent noradrenaline transporter	桃仁(2,3-didehydro GA70, hederagenin,), 红花(kaempferol, Stigmasterol), 夏枯草(kaempferol, Stigmasterol), 熟地黄(Stigmasterol)
Mianserin	Alpha-2A adrenergic receptor	红花(Stigmasterol), 三七(Stigmasterol), 丹参(4-methylenemiltirone,salviolone)
	Sodium-dependent noradrenaline transporter	桃仁(2,3-didehydro GA70, hederagenin), 红花(kaempferol, Stigmasterol), 白芍(kaempferol), 三七(Stigmasterol)
	Sodium-dependent serotonin transporter	桃仁(beta-sitosterol), 紫苏(beta-sitosterol), 红花(beta-sitosterol), 乌药(beta-sitosterol, Boldine) 白芍(beta-sitosterol), 藿香(5-Hydroxy-7,4′-dimethoxyflavanon), 丹参(1,2,5,6-tetrahydrotanshinone, 2-isopropyl-8-methylphenanthrene-3,4-dione, 4-methylenemiltirone, Methylenetanshinquinone, danshenspiroketallactone, Dihydrotanshinlactone, Epidanshenspiroketallactone, neocryptotanshinone ii,salviolone)
	Alpha-2C adrenergic receptor	丹参(4-methylenemiltirone, Miltirone,), 乌药(6,7-dimethoxy-2-(2-phenylethyl)chromone, DMPEC)
	Alpha-2B adrenergic receptor	丹参(salviolone)
	Sodium-dependent dopamine transporter	红花(Stigmasterol), 丹参(1,2,5,6-tetrahydrotanshinone, 2-isopropyl-8-methylphenanthrene-3,4-dione,4-methylenemiltirone,dihydrotanshinlactone, Miltirone, neocryptotanshinone ii, salviolone), 乌药(6,7-dimethoxy-2-(2-phenylethyl)chromone, DMPEC, Boldine)
Trazodone	Sodium-dependent serotonin transporter	桃仁(beta-sitosterol), 紫苏(beta-sitosterol), 红花(beta-sitosterol), 乌药(beta-sitosterol, Boldine) 藿香(5-Hydroxy-7,4′-dimethoxyflavanon), 丹参(1,2,5,6-tetrahydrotanshinone, 2-isopropyl-8-methylphenanthrene-3,4-dione, 4-methylenemiltirone, Methylenetanshinquinone, danshenspiroketallactone, Dihydrotanshinlactone, Epidanshenspiroketallactone, neocryptotanshinone ii,salviolone)
	Alpha-1A adrenergic receptor	桃仁(gibberellin 7), 红花(Stigmasterol), 丹参(1,2,5,6-tetrahydrotanshinone, sugiol, Dehydrotanshinone II A, 5,6-dihydroxy-7-isopropyl-1,1-dimethyl-2,3, 2-isopropyl-8-methylphenanthrene-3,4-dione,4-methylenemiltirone, 3-beta-Hydroxymethyllenetanshiquinone,Methylenetanshinquinone, przewaquinone c, cryptotanshinone, danshenspiroketallactone, deoxyneocryptotanshinone, dihydrotanshinlactone, dihydrotanshinoneI, epidanshenspiroketallactone, C09092, isocryptotanshi-none, Isotanshinone II, miltionone I, Miltirone, neocryptotanshinone ii, 1-methyl-8,9-dihydro-7H-naphtho[5,6-g]benzofuran-6,10,11-trione, salviolone, tanshinone iia, (6S)-6-(hydroxymethyl)-1,6-dimethyl-8,9-dihydro-7H-naphtho[8,7-g]benzofuran-10,11-dione,)
	Alpha-2A adrenergic receptor	红花(Stigmasterol), 丹参(4-methylenemiltirone,salviolone)
Nortriptyline	Sodium-dependent noradrenaline transporter	桃仁(2,3-didehydro GA70, hederagenin,), 红花(kaempferol, Stigmasterol), 白芍(kaempferol)
	Sodium-dependent serotonin transporter	桃仁(beta-sitosterol), 紫苏(beta-sitosterol), 红花(beta-sitosterol), 白芍(beta-sitosterol), 藿香(5-Hydroxy-7,4′-dimethoxyflavanon), 丹参(1,2,5,6-tetrahydrotanshinone, 2-isopropyl-8-methylphenanthrene-3,4-dione, 4-methylenemiltirone, Methylenetanshinquinone, danshenspiroketallactone, Dihydrotanshinlactone, Epidanshenspiroketallactone, neocryptotanshinone ii,salviolone)
	Alpha-1A adrenergic receptor	桃仁(gibberellin 7), 红花(Stigmasterol), 熟地黄(Stigmasterol), 丹参(1,2,5,6-tetrahydrotanshinone, sugiol, Dehydrotanshinone II A, 5,6-dihydroxy-7-isopropyl-1,1-dimethyl-2,3, 2-isopropyl-8-methylphenanthrene-3,4-dione,4-methylenemiltirone, 3-beta-Hydroxymethyllenetanshiquinone,Methylenetanshinquinone, przewaquinone c, cryptotanshinone, danshenspiroketallactone, deoxyneocryptotanshinone, dihydrotanshinlactone, dihydrotanshinoneI, epidanshenspiroketallactone, C09092, isocryptotanshi-none, Isotanshinone II, miltionone I, Miltirone, neocryptotanshinone ii, 1-methyl-8,9-dihydro-7H-naphtho[5,6-g]benzofuran-6,10,11-trione, salviolone, tanshinone iia, (6S)-6-(hydroxymethyl)-1,6-dimethyl-8,9-dihydro-7H-naphtho[8,7-g]benzofuran-10,11-dione,)
	Alpha-1D adrenergic receptor	丹参(1,2,5,6-tetrahydrotanshinone,Sugiol,2-isopropyl-8-methylphenanthrene-3,4-dione, 4-methylenemiltirone, cryptotanshinone, danshenspiroketallactone, neocryptotanshinone,) 乌药(Norboldine,Boldine)
Duloxetine	Sodium-dependent serotonin transporter	桃仁(beta-sitosterol), 紫苏(beta-sitosterol), 红花(beta-sitosterol), 乌药(beta-sitosterol, Boldine) 赤芍(beta-sitosterol), 藿香(5-Hydroxy-7,4′-dimethoxyflavanon), 丹参(1,2,5,6-tetrahydrotanshinone, 2-isopropyl-8-methylphenanthrene-3,4-dione, 4-methylenemiltirone, Methylenetanshinquinone, danshenspiroketallactone, Dihydrotanshinlactone, Epidanshenspiroketallactone, neocryptotanshinone ii,salviolone)
	Sodium-dependent noradrenaline transporter	桃仁(2,3-didehydro GA70, hederagenin,), 红花(kaempferol, Stigmasterol), 熟地黄(Stigmasterol), 三七(Stigmasterol), 赤芍(Stigmasterol)
	Sodium-dependent dopamine transporter	红花(Stigmasterol), 熟地黄(Stigmasterol), 赤芍(Stigmasterol), 丹参(1,2,5,6-tetrahydrotanshinone, 2-isopropyl-8-methylphenanthrene-3,4-dione,4-methylenemiltirone,dihydrotanshinlactone, Miltirone, neocryptotanshinone ii, salviolone), 乌药(6,7-dimethoxy-2-(2-phenylethyl)chromone, DMPEC,Boldine)
Clomipramine	Sodium-dependent serotonin transporter	桃仁(beta-sitosterol), 紫苏(beta-sitosterol), 红花(beta-sitosterol), 夏枯草(beta-sitosterol), 乌药(beta-sitosterol, Boldine), 黄柏(beta-sitosterol, Cavidine, Fumarine, Isocorypalmine, (S)-Canadine), 白芍(beta-sitosterol), 藿香(5-Hydroxy-7,4′-dimethoxyflavanon), 丹参(1,2,5,6-tetrahydrotanshinone, 2-isopropyl-8-methylphenanthrene-3,4-dione, 4-methylenemiltirone, Methylenetanshinquinone, danshenspiroketallactone, Dihydrotanshinlactone, Epidanshenspiroketallactone, neocryptotanshinone ii,salviolone)
	Sodium-dependent noradrenaline transporter	桃仁(2,3-didehydro GA70, hederagenin,), 红花(kaempferol, Stigmasterol), 白芍(kaempferol), 熟地黄(Stigmasterol)
	Glutathione S-transferase P	藿香(quercetin), 红花(quercetin, luteolin, kaempferol), 乌药(quercetin), 紫苏(luteolin), 丹参(luteolin), 白芍(kaempferol)

## Discussions

In this study, we extracted drug and disease information from the PubMed records, and developed an algorithm to identify the drug with obvious side effects to be replaced. The indications of those drugs were translated and searched in the CNKI to extract herb and disease relationships. We also developed an algorithm to calculate the substitutability of target drugs and target herbs by considering the proportion of herb in the TCM prescriptions to the conditions for which the drug is indicated, and the side effects produced by the drug. The algorithms themselves are rather simple, but as shown in the case study of depression drug alternatives, the proposed approach could sort out viable candidates from multiple sources of biomedical databases across the western and Chinese literature. Our case study of depression, identified 10 alternative herbs, ranked by our method for each candidate drug. We chose the drug with the highest side effects and its related herb to evaluate our methods. The graph of Herb-Ingredients-Target-Disease relations shows the herbs of similarly curative effect of the western medicine can reduce the side-effects at the same time. The protein-protein interaction network implied that alternative herbs may help the western drug to improve the effectiveness of the treatment at the gene or protein level. Further, the gene enrichment analysis shows the enriched Gene Ontology terms for biological process, cellular component, and molecular function, and KEGG Pathways of targets from the main active ingredients of alternative herbs. With respect to biological process, the following two pathways are ranked in top 10: the adenylate cyclase-activating adrenergic receptor signaling pathway (GO:0071880) and the adrenergic receptor signaling pathway (GO:0071875). With the adenylyl cyclase being activated, GO:0071880 proceeds to increase the cyclic adenosine monophosphate (CAMP) concentration (https://www.ebi.ac.uk/QuickGO/term/GO:0071880). CAMP is known as a second messenger in cellular activity [33]. It responds the binding of an extracellular signal to a receptor on the cell surface by its concentration, and regulates the activity of intracellular enzymes and non-enzymatic proteins to carry and amplify signals in cell signal transduction pathway. Adrenaline is used to treat hypertension and asthma, which are side effects of Nefazodone. In cellular component, the GABA-A receptor complex (GO:1902711) ranked in top 10. Once GABA binds to the receptor, the receptor changes its configuration on the cell membrane. Then, with the open channel hole, chloride anions can be allowed to pass down an electrochemical gradient. GABAA receptor is triggered and can create a more stable environment for the resting potential. Moreover, it can make the cell hyperpolarization which weakens the depolarization influence of the excitatory neurotransmitter and decrease the possibility of producing action potential. Thus, the role of this receptor is to inhabit and reduce the activity of neuron [34]. GABA-A contributes to receptor activation, and has anxiolytic, anticonvulsant, amnesic, sedative, hypnotic and euphoric properties. In terms of molecular function, G-protein coupled neurotransmitter receptor activity (GO:0099528) is ranked top 3. The G protein coupled receptor (GPCR) promotes an associated G protein by catalyzing the exchange of GDP to GTP. This process causes the G protein to be activated and participate in the next step of signaling. The receptor for serotonin is a G-protein-coupled receptor [35]. KEGG Pathways show that the targets of the main active ingredients of alternative herbs mainly act on the AGE-RAGE signaling pathway, which is important in in diabetic complications, cancer, and hepatitis.

## Conclusions

Previous studies into the relationship between drugs and herbs were based on network construction. However, most of the studies did not consider the proportion of the herb dosage in the prescription and the side effect produced by the target drug for drug and herb relation ranking. In this study, we utilized not only Western biomedical databases, but also the TCM literature. The results indicated that the top-ranked herbs were highly related to the target indication, depression, and could mitigate the side-effects of the target drug, which could help in the development of new drugs. In the future, we intend to conduct an in-depth analysis of target drugs and alternative herbs. We will include the similarity of chemical compounds into our method.

## Supplementary information

**Additional file 1.** Whole indication name collection.

## Data Availability

All data generated during this study are included in this published article.

## Abbreviations

TCM: Traditional Chinese Medicine
CKNI: China National Knowledge Internet
ADR: Adverse drug reactions
SADRs: Serious adverse drug reactions
CAM: Complementary and Alternative Medicine
TCMID: Traditional Chinese medicine integrative database
TCMSP: Traditional Chinese medicine systems pharmacology database
PPI: Protein-protein interaction
HIT: Herb Ingredients’ Targets
SVMs: Support vector machines
NER: Named Entity Recognition
TTD: Therapeutic Target Database
CTPD: Chinese Traditional Prescriptions Database
CAMP: Cyclic adenosine monophosphate
GPCR: G protein coupled receptor
