# Supplementary material for: Literature based discovery of alternative TCM medicine for adverse reactions to depression drugs
Source: BMC Bioinformatics. 2020 Oct 26;21(Suppl 5):405. doi: 10.1186/s12859-020-03735-8 (PMC7586667; doi:10.1186/s12859-020-03735-8)
Supplement: Supplementary file 1 — Additional file 1. Whole indication name collection. [file 12859_2020_3735_MOESM1_ESM.docx]

Additional file 1. Whole indication name collection

| Rank | Indication name | | No. of Papers | Rank | Indication name | | No. of Papers |
| --- | --- | --- | --- | --- | --- | --- | --- |
|  | English | Chinese |  |  | English | Chinese |  |
| 1 | arthritis | 关节炎 | 5307 | 34 | essential hypertension | 原发性高血压 | 193 |
| 2 | pain | 疼痛 | 3578 | 35 | primary hypertension | 原发性高血压 | 193 |
| 3 | diabetes | 糖尿病 | 3554 | 36 | malaria | 疟疾 | 189 |
| 4 | hypertension | 高血压 | 3008 | 37 | bacterial infections | 细菌感染 | 170 |
| 5 | asthma | 哮喘 | 2911 | 38 | schizophrenia | 精神分裂症 | 133 |
| 6 | blood pressure | 血压 | 2472 | 39 | acute pain | 疼痛 | 120 |
| 7 | lung cancer | 肺癌 | 2207 | 40 | urinary tract infections | 尿路感染 | 93 |
| 8 | breast cancer | 乳腺癌 | 1690 | 41 | hypotension | 低血压 | 93 |
| 9 | heart failure | 心力衰竭 | 1674 | 42 | chronic obstructive pulmonary disease | 慢性阻塞性肺病 | 86 |
| 10 | major depression | 抑郁症 | 1618 | 43 | ocular disease | 眼病 | 83 |
| 11 | rheumatoid arthritis | 类风湿性关节炎 | 1541 | 44 | acute migraine | 急性偏头痛 | 73 |
| 12 | pneumonia | 肺炎 | 1428 | 45 | erectile dysfunction | 勃起功能障碍 | 68 |
| 13 | diabetic nephropathy | 糖尿病性肾病 | 1305 | 46 | allergic conjunctivitis | 过敏性结膜炎 | 65 |
| 14 | diarrhea | 腹泻 | 1303 | 47 | atrial fibrillation | 心房颤动 | 56 |
| 15 | constipation | 便秘 | 1243 | 48 | respiratory failure | 呼吸衰竭 | 20 |
| 16 | seizures | 癫痫 | 1073 | 49 | acute asthma | 急性哮喘 | 18 |
| 17 | acne vulgaris | 寻常痤疮 | 948 | 50 | arrhythmia | 心律不齐 | 15 |
| 18 | angina pectoris | 心绞痛 | 927 | 51 | excessive bleeding | 大出血 | 9 |
| 19 | insomnia | 失眠 | 827 | 52 | viral infections | 病毒性感染 | 8 |
| 20 | diarrhea | 痢疾 | 796 | 53 | tachyarrhythmias | 心律加快 | 6 |
| 21 | bronchial asthma | 支气管哮喘 | 740 | 54 | depression | 忧郁 | 3 |
| 22 | ascites | 腹水 | 676 | 55 | intermittent claudication | 间歇性跛行 | 3 |
| 23 | migraine headaches | 偏头痛 | 591 | 56 | chronic angina | 慢性心绞痛 | 2 |
| 24 | myocardial infarction | 心肌梗塞 | 591 | 57 | pulmonary hypertension | 肺动脉高血压 | 2 |
| 25 | thrombocytopenia | 血小板减少 | 583 | 58 | venous thromboembolism | 静脉血栓栓塞 | 2 |
| 26 | anxiety disorder | 焦虑症 | 475 | 59 | major depressive disorder | 抑郁症 | 1 |
| 27 | pulmonary arterial hypertension | 肺动脉高压 | 467 | 60 | hypercalcemia | 高钙血 | 1 |
| 28 | ovarian cancer | 卵巢癌 | 422 | 61 | chronic idiopathic constipation | 慢性特发性便秘 | 0 |
| 29 | cancer | 癌症 | 421 | 62 | metastatic disease | 转移性疾病 | 0 |
| 30 | congestive heart failure | 充血性心力衰竭 | 303 | 63 | mild persistent asthma | 轻度持续性哮喘 | 0 |
| 31 | pancreatic cancer | 胰腺癌 | 263 | 64 | orthostatic hypotension | 起立性低血压 | 0 |
| 32 | acute heart failure | 急性心力衰竭 | 242 | 65 | sleep maintenance insomnia | 睡眠维护失眠 | 0 |
| 33 | fungal infections | 真菌感染 | 214 | 66 | worm infections | 蠕虫病毒感染 | 0 |
